# Discovery, Validation, and Target Prediction of Antibacterial and Antidiabetic Components of *Archidendron clypearia* Based on a Combination of Multiple Analytical Methods

**DOI:** 10.3390/molecules28031329

**Published:** 2023-01-30

**Authors:** Wenduo Ji, Lixia Gu, Xuezhe Zou, Zhichao Li, Xiaohong Xu, Jialin Wu, Shu Zhang, Hong Deng

**Affiliations:** 1 The Center for Drug Research and Development, Guangdong Provincial Key Laboratory of Advanced Drug Delivery Systems, Guangdong Pharmaceutical University, Guangzhou 510006, China; 2 School of Pharmaceutical Sciences (Shenzhen), Shenzhen Campus of Sun Yat-sen University, Shenzhen 518107, China; 3 Guangdong Provincial Engineering Center of Topical Precise Drug Delivery System, Department of Pharmaceutics, Guangdong Pharmaceutical University, Guangzhou 510006, China

**Keywords:** *Archidendron clypearia*, spectrum–effect relationship, antibacterial, antidiabetic, network pharmacology, molecular docking

## Abstract

*Archidendron clypearia* (*A. clypearia*), a Fabaceae family member, is widely used as an anti-inflammatory herbal medicine; however, its antibacterial and antidiabetic properties have not been extensively investigated. This study aimed to systematically analyze the antibacterial and antidiabetic components of *A. clypearia* by utilizing a combination of analytical methods. First, ten different polarity extracts were analyzed through ultra-performance liquid chromatography (UPLC), and their antibacterial and antidiabetic activities were evaluated. Then the spectrum–effect relationship between the biological activity and UPLC chromatograms was analyzed by partial least squares regression and gray relational analysis, followed by corresponding validation using isolated components. Finally, network pharmacology and molecular docking were implemented to predict the main antibacterial target components of *A. clypearia* and the enzyme inhibition active sites of α-amylase and α-glucosidase. P15, P16, and P20 were found to be the antibacterial and antidiabetic active components. The inhibitory effect of 7-O-galloyltricetiflavan (P15) on six bacterial species may be mediated through the lipid and atherosclerosis pathway, prostate cancer, adherens junctions, and targets such as SRC, MAPK1, and AKT1. The molecular docking results revealed that 7-O-galloyltricetiflavan and 7,4′-di-O-galloyltricetiflavan (P16/P20) can bind to α-amylase and α-glucosidase pockets with binding energies lower than −6 kcal/mol. Our study provides guidance for the development of antibacterial and antidiabetic products based on *A. clypearia* and can be used as a reference for the evaluation of bioactivity of other herbs.

## 1. Introduction

*Archidendron clypearia (Jack.)* Nielsen (*A. clypearia*), a member of the Fabaceae family, has been widely used as a traditional medicine for detoxification, cooling, and edema reduction in Southeast Asia because it is composed of abundant polyphenols [1]. The Chinese folklore book “Lu Chuan Ben Cao” records the use of *A. clypearia* for the treatment of burns and ulcers since the 17th century. Moreover, *A. clypearia* is reported to have various effects, including anti-inflammatory, antioxidant, antibacterial, and antidiabetic effects. However, its exploitation is mostly based on its overall extract, and the specific efficacy of the components of *A. clypearia* has not been extensively investigated.

*Salmonella*, *Bacillus subtilis*, *Klebsiella pneumoniae*, *Staphylococcus aureus*, *Escherichia coli* and *Pseudomonas aeruginosa* are common pathogens that cause diseases such as diarrhea, fever, and pneumonia, and can even endanger human life [2]. *A. clypearia* is rich in polyphenols that have been widely demonstrated to have antibacterial effects. The common antibacterial mechanisms of polyphenols mainly include cell wall disruption, alteration of cell membrane permeability, cell metabolism changes, and DNA synthesis disruption [3]. The effects of different components of polyphenols on different pathogenic bacteria also vary greatly. Thus, the search for highly active polyphenolic components is significant for promoting the development of antibacterial products.

**Figure 1:**
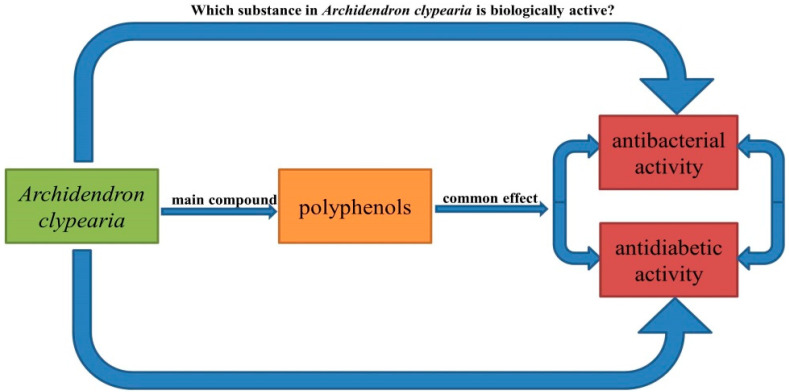
Correlation between *A. clypearia* and the selected targets.

Diabetes mellitus (DM) is a group of clinical syndromes caused by the interaction of genetic and environmental factors. While DM has a complex etiology, it is closely related to the dysregulation of α-glucosidase and α-amylase activity, and the determination of the inhibitory activity of drugs toward these two enzymes is an important indicator of their hypoglycemic ability [4]. Although traditional antidiabetic drugs such as acarbose and metformin are highly effective, they have various side effects including flatulence, indigestion and other gastrointestinal reactions, whereas many herbal medicines have low toxicity and a wide range of efficacy. Therefore, novel antidiabetic drugs based on herbal medicines can be used to overcome the limitations of the current antidiabetic drugs, provide a more diverse choice of treatments, and improve the standard of living of diabetic patients (Figure 1).

The traditional method for screening active substances in natural products is multistep extraction and separation using organic solvents followed by activity determination. However, this method is time-consuming, labor-intensive, environmentally unfriendly, and inefficient. Spectrum–effect relationship analysis is a method for determining active ingredients by correlating the content of the product in the spectrum of the research object with their bioactivity via chemometrics [5]. Spectrum–effect relationship analysis can be carried out on different extracts or extraction sites of the same herb. Spectrum–effect relationship analysis has been widely used in the discovery for active ingredients of various herbal medicines.

Network pharmacology was first proposed by Hopkins in 2007 [6]. It is based on the theory of systems biology and integrates the techniques of multiple disciplines such as multidirectional pharmacology, bioinformatics, and computer science to construct a multilevel “disease–target–drug” network in order to explore the correlation between drugs and diseases and elucidate the mechanism of drug action. Network pharmacology has holistic and systematic characteristics that overlap with the multicomponent and multi-action characteristics of Chinese herbal ingredients. Currently, the mechanism of the active components of *A. clypearia* is poorly understood. We used a combination of network pharmacology and spectrum–effect relationship analysis to construct a “component–target–disease” network for determining the potential active components of *A. clypearia*, employing tools such as topological parameter analysis, visualization of protein interaction network diagrams, histograms, bubble diagrams, and drug action on target sites to predict the mechanism of action.

Molecular docking is the process of finding the optimal binding mode between small molecules (ligands) and biomolecules (receptors) by simulating the geometric and energetic matching of molecules through chemometric methods, and includes the rigid, semi-flexible, and flexible docking methods. Molecular docking can be used to simulate the binding site between a drug molecule and its corresponding ligand and to assess its energy of action; it is beneficial for guiding drug development and elucidating the mechanism of action of a drug [7].

**Figure 2:**
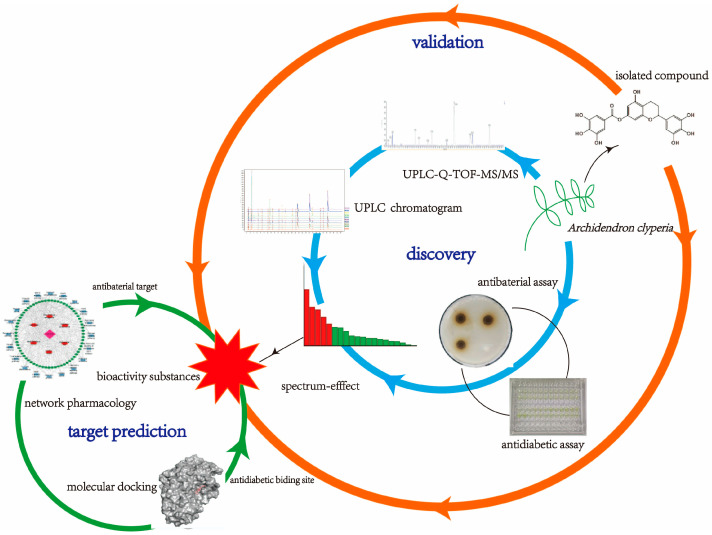
Strategy for studying the antibacterial and antidiabetic components extracted from *A. clypearia*.

In this study, the main components of 70% aqueous ethanol (EtOH) extracts of *A. clypearia* were identified, and the spectrum–effect relationship between the biological activity and UPLC chromatograms was analyzed through partial least squares regression (PLSR) and gray relational analysis (GRA). The screening results were validated through pharmacological activity testing of isolated compounds. The mechanism of action of the screened antibacterial substances was also predicted and evaluated by network pharmacology. Furthermore, the enzyme inhibition active sites of α-amylase and α-glucosidase were analyzed by molecular docking. The combination of multiple analytical methods including spectrum–effect relationship analysis, network pharmacology, and molecular docking allows the exploitation of the medicinal value of *A. clypearia* (Figure 2).

## 2. Results and Discussion

### 2.1. Characterization of A. clypearia

The S1 extract was analyzed by mass spectroscopy. Based on the relative retention time, mass-to-charge ratio (*m*/*z*), number of fragment ions, and literature references, the components of *A. clypearia* were analyzed, and 26 compounds were identified. The detailed data attribution is described in Table 1, and the total ion current is shown in Figure S1.

### 2.2. UPLC Chromatographic Analysis of Extracts

**Figure 3:**
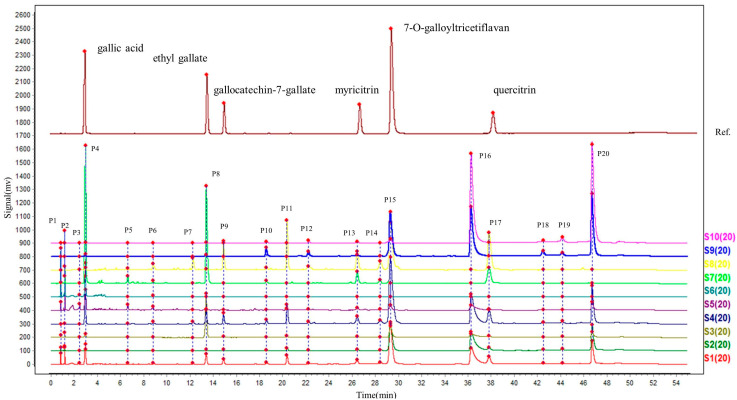
UPLC chromatograms of the different polarity extracts of *A. clypearia* and standard solution.

A full wavelength scan (210–400 nm) of the test samples was performed during the method exploration stage, and a wavelength of 270 nm was chosen because it had a smooth baseline and the best response. Additionally, 70% ethanol was selected as the solvent for further analysis because of better sample stability after a comparison to methanol, 50% methanol, and 30% ethanol. As shown in Figure 3, 20 peaks representing more than 90% of the total peak area for S1, which can be easily separated and each have a large area, were selected as the characteristic peaks. The standard solution and the multipoint calibration function of the Similarity Evaluation System for Chromatographic Fingerprints of Traditional Chinese Medicines (Version 2012A; Beijing, China) were used to align the peaks of other extracts with those of S1. The time window was set to 0.2, the median method was applied, and the full spectrum peaks were matched after multipoint calibration. The quantified peak areas of the 20 characteristic peaks of the different polarity extracts were obtained (Table S1), the peak areas of the components with undetected peak areas were set to 0.01, and the peak areas of each peak were normalized to the total peak area for further analysis.

The validated results of the 20 characteristic peaks are summarized in Table S2. All relative standard deviation (RSD) values of the normalized peak areas were less than 5%.

### 2.3. Antibacterial Activity of Extracts

The results for the analysis of the antibacterial activity of the different polarity extracts are shown in Table 2 and Figure S2. Overall, the different polarity extracts show different degrees of inhibition of the six bacteria. The ethyl acetate (S4) and 80% ethanol extracts (S10) showed the highest antibacterial activity with antibacterial zone diameters greater than 10 mm, while the aqueous extract (S6) and 20% ethanol extract (S7) exhibited low antibacterial activity, with antibacterial zone diameters below 10 mm. The standard antibiotic gentamicin exhibited the highest inhibition against the tested bacteria. This observation was in agreement with the results of Liang et al. [8], who reported that flavonoids and organic acids affect bacteria by disrupting bacterial cell walls, inhibiting the synthesis of key bacterial proteins, and by interfering with bacterial DNA replication [9]. In our study, the large flavanone molecules affected bacterial activity more strongly than the small organic acid molecules, which may indicate that large flavanone molecules have a higher fit with bacteria-related enzyme systems.

MIC experiments were conducted to corroborate the abovementioned assay results; the results are summarized in Table S3. The different polarity extracts exhibited a relatively weak inhibitory effect on *Salmonella* and *Staphylococcus aureus*, with MIC values above 0.78 mg/mL, while the inhibitory effect on *Escherichia coli* was high, with MIC values above 0.2 mg/mL. Overall, the ethyl acetate (S4) and 80% ethanol (S10) extracts exhibited the highest antibacterial activity. The antibacterial zone diameter values and MIC results were generally consistent.

### 2.4. α-Glucosidase and α-Amylase Inhibition Assay

**Figure 4:**
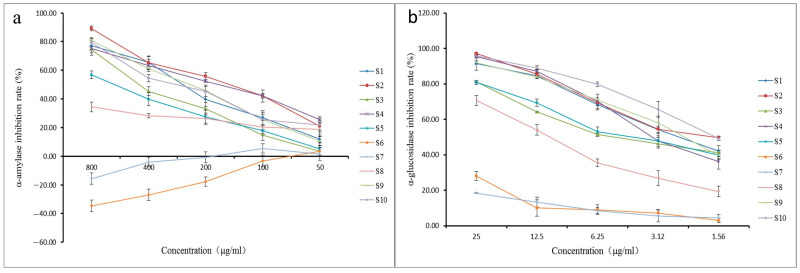
α-Amylase and α-glucosidase inhibition rates of different polarity extracts of *A. clypearia*. (**a**) Inhibition rate of α-amylase; (**b**) inhibition rate of α-glucosidase.

The inhibition rate results are shown in Figure 4. With the exception of S6 and S7, all extracts showed stronger α-amylase inhibition with increasing concentration. S6 and S7 partially interfered with the determination of the α-amylase inhibitory activity owing to the presence of certain sugar components in the samples, and the inhibition rate became negative with increasing sample concentration. The α-glucosidase inhibition rates of different extracts of *A. clypearia* increased with increasing concentrations, with S2, S9, and S10 exhibiting the strongest α-glucosidase inhibition.

The IC_50_ values of the samples for enzyme inhibition are listed in Table 3. Here, a smaller IC_50_ value indicates a stronger enzyme inhibitory effect. The order of the IC_50_ values of the different extracts was S8 > S5 > S3 > S10 > S1 > S9 > S4 > S2, and the order of the α-amylase inhibition ability was S2 > S4 > S9 > S1 > S10 > S3 > S5 > S8. The inhibition rates of S6 and S7 were too low to determine their IC_50_ values. Similarly, the order of the α-glucosidase inhibition ability of the different polarity extracts was S10 > S2 > S9 > S1 > S4 > S5 > S3 > S8 > S6 > S7.

### 2.5. Spectrum–Effect Relationship Analysis Results

#### 2.5.1. GRA Results

The correlation results for the different polarity extracts of *A. clypearia* with the six bacteria and two hypoglycemia-related enzymes are shown in Table 4. Among the 20 characteristic peaks, P15 is the best antibacterial activity component owing to its high correlation (>0.8) and is ranked in the top two for the six model bacteria. The other components ranked in the top six as the antibacterial substances are P10, P12, P14, P16, P18, and P20. In the α-amylase and α-glucosidase GRA models, P1, P3, P15, P16, P18, P19, and P20, which have correlations in the 0.8–0.9 range, are the main enzyme inhibition components of *A. clypearia* [10]. The results show that the *A. clypearia* extracts with low polarities mainly exhibit antibacterial activity and enzyme inhibitory activity.

#### 2.5.2. PLSR Results

**Figure 5:**
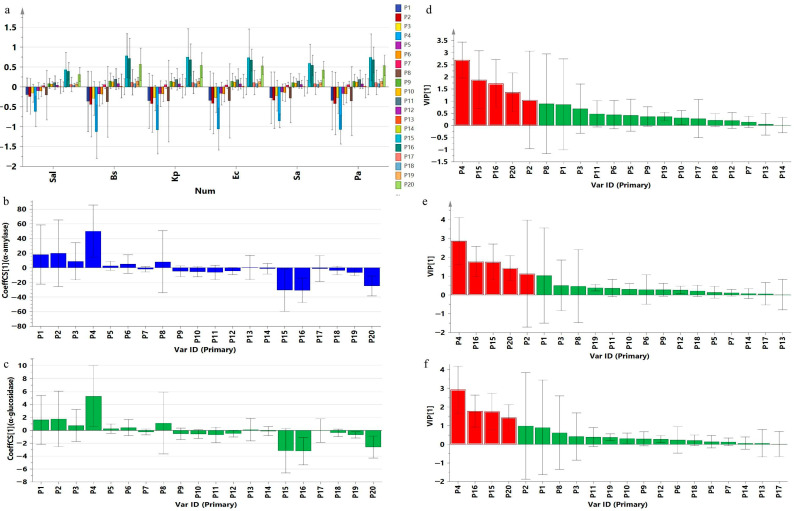
Results of the PLS model analysis. (**a**) Coefficient plot of six bacteria; (**b**) coefficient plot of α-amylase; (**c**) coefficient plot of α-glucosidase; (**d**) VIP plot of six bacteria; (**e**) VIP plot of α-amylase; (**f**) VIP plot of α-glucosidase.

The six bacteria and two enzymes were evaluated by the PLS model; the results are shown in Figure 5. The Q^2^ and R^2^ values of the established model are greater than 0.5, indicating that the established model is accurate. It was observed from the correlation plot (Figure 5a–c) that the effect of the different polarity extracts on the six bacteria is the same as that on the two enzymes. The diameter of the antibacterial zone is negatively correlated with the components in the first half of the UPLC gradient and is positively correlated with the components in the second half, indicating that the inhibitory effect of the effective components is mostly associated with the low-polarity components. Furthermore, the VIP values indicate the magnitude of the inhibitory activity for the six bacteria and two hypoglycemia-related enzymes. The VIP values of P15, P16, and P20 are greater than one and are positively correlated with the diameter of the antibacterial zone; thus, P15, P16, and P20 are considered to contain antibacterial and antidiabetic components [11].

In summary, combining the results of the abovementioned two analytical methods, it can be confirmed that P15 shows a good correlation with the inhibition of the six bacteria and two enzymes, and is identified as a compound containing bioactive substances; thus, P15 can be used for further pharmacological research and product development. Moreover, P16 and P20 exhibit high α-glucosidase and α-amylase inhibitory activity, and can be used for further analysis.

### 2.6. Verification of Active Compounds

Six compounds were isolated for the verification of antibacterial and antidiabetic activity, and the spectroscopic data of the isolated compounds are provided in the Supplementary Materials. Gallic acid (P4), ethyl gallate (P8), myricitrin (P14), and quercitrin (P17) were identified by comparison with the standard substances, while gallocatechin-7-gallate (P9) and 7-O-galloyltricetiflavan (P15) were identified by comparison of the mass spectra, ^1^H NMR spectra, and ^13^C NMR spectra data for these compounds to those of the reported substances. These six components can be further categorized as organic acids (P4, P8), flavanones (P9, P15), and flavonoids (P14, P17).

The MIC values of the isolated substances for six bacteria were determined; the results are shown in Table 5. The order of the MIC values of the six components for the inhibition of the six bacteria is flavonoids < organic acids < flavanones. Among these, P9 and P15 exhibit higher inhibitory activity, which is consistent with the results of the spectrum–effect relationship analysis. Although the MIC values of 7-O-galloyltricetiflavan were relatively higher compared to gentamicin, they were less than 0.2. Thus, 7-O-galloyltricetiflavan was considered the main compound with antibacterial activity.

**Figure 6:**
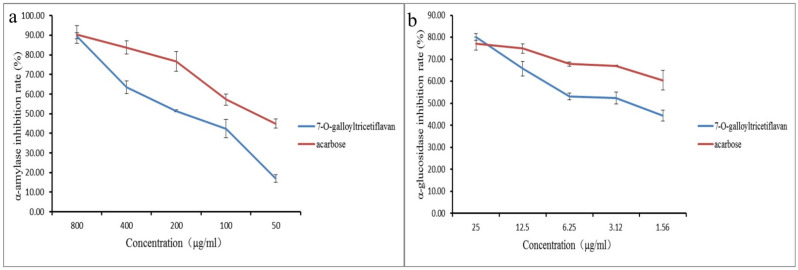
α-Amylase and α-glucosidase inhibition rates of 7-O-galloyltricetiflavan and acarbose. (**a**) Inhibition rate of α-amylase; (**b**) inhibition rate of α-glucosidase.

The IC_50_ values of the six isolated compounds for α-glucosidase and α-amylase are shown in Table S4. The flavanones are more effective than the organic acids and flavonoids toward the inhibition of both enzymes. Compared to the positive control acarbose, P15 shows a low value in the α-amylase inhibition assay. In contrast, the α-glucosidase inhibition curves reveal that acarbose exhibits stronger inhibition at low concentrations, whereas P15 exhibits higher enzyme inhibitory activity at high concentrations (Figure 6). It has been reported that acarbose typically causes side effects such as gastrointestinal flatulence, intestinal sounds, abdominal pain, bloating, and diarrhea, which may be due to the strong inhibitory effect of acarbose on α-amylase, resulting in the accumulation of undigested starch in the intestine and the release of gas [12]. P15 is less active than acarbose in inhibiting α-amylase, but allows stronger α-glucosidase inhibition in a certain concentration range; thus, P15 can be developed as a hypoglycemic drug to overcome the problem of the side effects of acarbose.

### 2.7. Prediction of Antibacterial Targets

**Figure 7:**
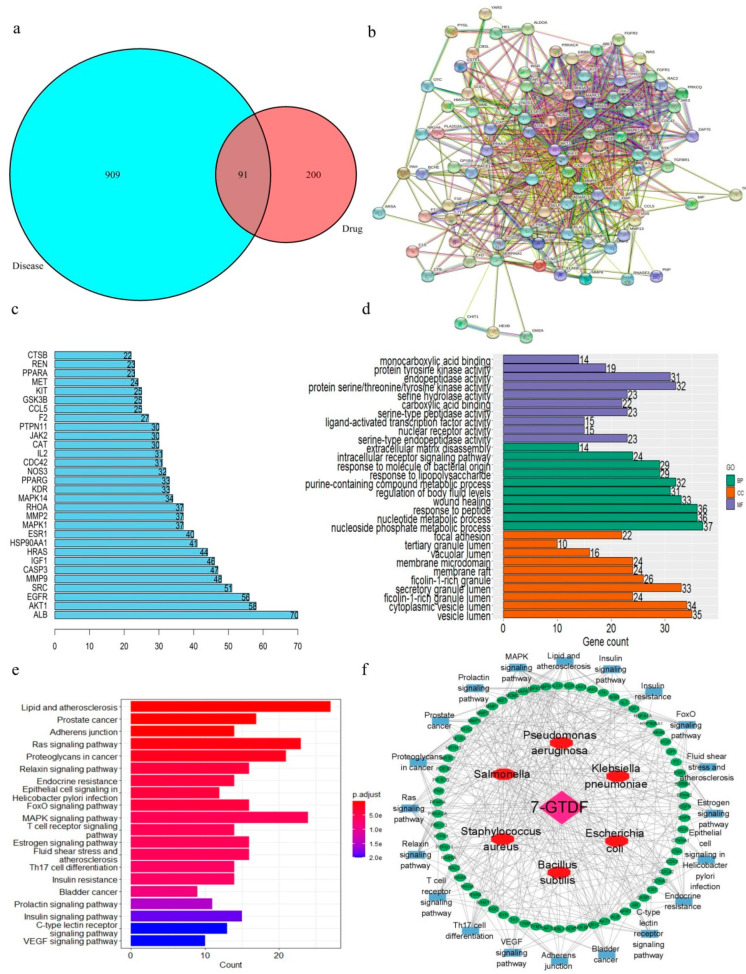
Prediction and analysis of the antibacterial mechanism of 7-O-galloyltricetiflavan. (**a**) Venn diagram of differential chemical markers and intersection targets; (**b**) PPI network; (**c**) node degree bar plot; (**d**) GO functional enrichment analysis; (**e**) KEGG pathway enrichment analysis; (**f**) component–target–pathway–disease network.

7-O-Galloyltricetiflavan (P15), which is the compound exhibiting the highest-screened antibacterial activity and is the most abundant compound in *A. clypearia*, was used to study potential antibacterial targets through network pharmacology. A set of 223 overlapping components and disease targets was obtained by Venn diagrams (Figure 7a). The PPI network was further constructed successfully by the STRING platform with 223 nodes, 493 edges, and an average degree value of 4.4 (Figure 7b). A higher degree value of the PPI network indicates a greater significance of the target [13]. The top 10 targets ranked in terms of nodes were SRC, MAPK1, AKT1, HSP90AA1, HRAS, PTPN11, EGFR, LCK, RHOA, and MAPK8 (Figure 7c).

The biological functions and signaling pathways involving the corresponding target proteins of 7-O-galloyltricetiflavan were determined by the GO and KEGG enrichment analyses, and the enrichment results were ranked by the *p*-value [14]. Overall, the bar graph of the GO analysis results shows that 7-O-galloyltricetiflavan mainly affects the nucleoside phosphate metabolism of biological processes, the vesicle lumen of cellular components, and the serine-type endopeptidase activity of molecular functions to exert its antibacterial effect (Figure 7d). Meanwhile, 7-O-galloyltricetiflavan mainly affected signaling pathways such as the lipid and atherosclerosis pathway, prostate cancer, and the adherens junction (Figure 7e). The lipid and atherosclerosis pathway was associated with inflammatory diseases such as coronary artery disease, myocardial infarction, and stroke, with a total of 27 targets being enriched. Prostate cancer and adherens junction pathways were strongly related to cancer. Thus, 7-O-galloyltricetiflavan exerted bacterial inhibitory effects because of the cross-talk of multiple in vivo signaling pathways.

Furthermore, the “component–target–pathway–disease” network of 7-O-galloyltricetiflavan was constructed using the PPI and KEGG results (Figure 7f). The network demonstrates the interaction relationships of 20 pathways and 67 key targets between 7-O-galloyltricetiflavan and six bacteria and will provide guidance for the discovery and validation of the antibacterial mechanism of 7-O-galloyltricetiflavan.

### 2.8. Molecular Docking Study

The inhibition of α-glucosidase and α-amylase by the main components was simulated by molecular docking. Prior to the molecular docking, grid boxes were designed for the combination of proteins and molecules according to the position of the ligands present in the original protein and as reported in the literature [15,16]. The docking box size parameters were x = 15.75, y = 15, and z = 16.5 for 1OSE and x = 16.5, y = 21.75, and z = 22.5 for 3A4A, while the position parameters were x = 37.522, y = 38.049, and z = −1.869 for 1OSE, and x = −12.213, y = −8.893, and z = 13.995 for 3A4A. The binding energies of the protein to the target molecules are shown in Table 6. The crystallized ligands were docked to assess the accuracy of the procedure, and the results showed that the binding energy of α-glucosidase to the crystallized ligand (α-d-glucose) was −5.20 kcal/mol with a root mean square deviation (RMSD) value of 0.538, and the binding energy of α-amylase to the crystallized ligand (β-d-glucose) was −5.07 kcal/mol with a RMSD value of 0.421, which further indicated the reliability of the docking results. A lower binding energy indicates a stronger effect of the interaction between the molecules and proteins. The order of the binding energy of the seven identified components to α-amylase was flavanones < flavonoids < organic acids, and the order of the binding energy to α-glucosidase is flavanones ≈ flavonoids < organic acids. The binding energies of the flavanones were all lower than −6 kcal/mol, indicating that they were more likely to interact with α-glucosidase and α-amylase [17].

**Figure 8:**
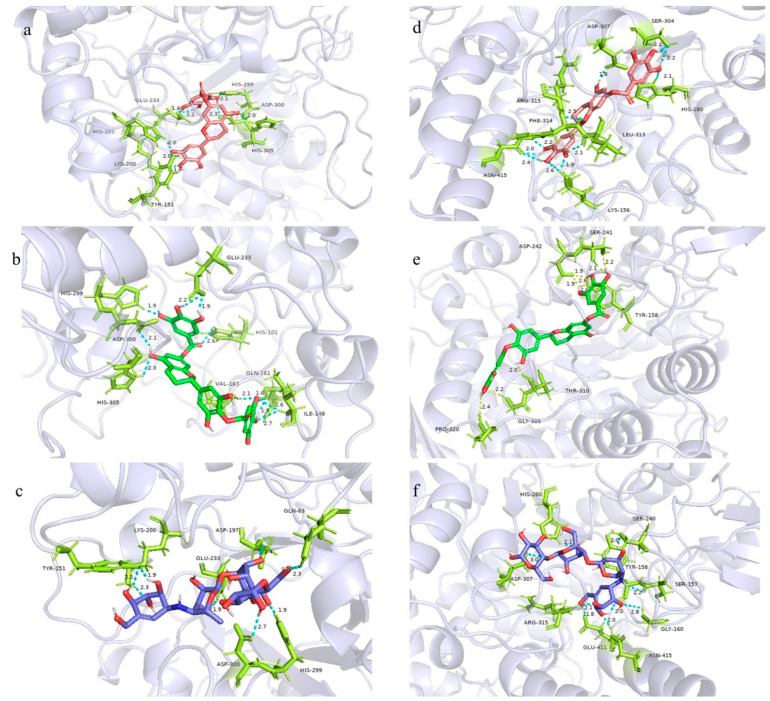
Molecular docking binding site of α-amylase (**a**) 7-O-galloyltricetiflavan; (**b**) 7,4′-di-O-galloyltricetiflavan; (**c**) acarbose; molecular docking binding site of α-glucosidase (**d**) 7-O-galloyltricetiflavan; (**e**) 7,4′-di-O-galloyltricetiflavan; (**f**) acarbose.

The specific binding sites of the major antidiabetic component and acarbose to the enzyme were further analyzed using PyMOL 2.4.0; the results are shown in Figure 8. Molecules 7-O-galloyltricetiflavan, 7,4′-di-O-galloyltricetiflavan, and acarbose showed similar binding sites, but with different specific amino acid residues. For α-amylase, the A ring of 7-O-galloyltricetiflavan is connected to the HIS-305 amino acid, the B ring is connected to the TYR-151 and HIS-201 amino acids; the 7-galloyl group is connected to the HIS-299, HIS-305, GLU-233 amino acid groups through hydrogen bonds. For α-glucosidase, the protein binds to the A ring of 7-O-galloyltricetiflavan through the ASP-307 amino acid, to the B ring through the LYS-156, LEU-313, PHE-314, and ASN-415 amino acids, to the C ring amino acid through ARG-315, and to the 7-galloyl group through HIS-280 and SER-304. Similarly, 7,4′-di-O-galloyltricetiflavan mainly combines with the HIS-101, ILE-148, GLN-161, VAL-163, GLU-233, HIS-299, ASP-300, and HIS-305 amino acid groups on α-amylase and with the TYR-158, SER-241, ASP-242, GLY-309, THR-310, and PRO-320 amino acid groups on α-glucosidase. The common binding amino acids of 7-O-galloyltricetiflavan and 7,4′-di-O-galloyltricetiflavan are most likely the critical sites in the antidiabetic process. In addition to hydrogen bonding with amino acids, hydrophobic bonding of the benzene ring, ionic bonding, and Van der Waals interactions contribute to the binding between proteins and molecules. Because of the presence of multiple bonding types and a perfect combination of the molecular structure in specific pockets on the proteins, antidiabetic effects are observed.

## 3. Materials and Methods

### 3.1. Reagents and Materials

#### 3.1.1. Plant Material

Dried leaves of *A. clypearia* were collected from Guangdong, Huizhou, China, in 2020 and were identified by Prof. Hanjing Yan at the School of Traditional Chinese Medicine, Guangdong Pharmaceutical University. Voucher specimens (202012001) are preserved in The Center for Drug Research and Development, Guangdong Pharmaceutical University.

#### 3.1.2. Other Reagents and Materials

Standard gallic acid, ethyl gallate, quercetin, and myricitrin compounds were purchased from Chengdu DST Biotechnology Co., Ltd. (Chengdu, China). The molecules 7-O-galloyltricetiflavan and gallocatechin-7-gallate were isolated from *A. clypearia* in our laboratory. *Salmonella* (CMCC 50115), *Bacillus subtilis* (ATCC 6633), *Klebsiella pneumoniae* (ATCC 13883), *Staphylococcus aureus* (ATCC 6538), *Escherichia coli* (ATCC 25922), and *Pseudomonas aeruginosa* (ATCC 27853) were supplied by the Guangdong Microbial Culture Collection Center. α-Glucoside (S10050-100UN), α-amylase (S31320-50k), and p-nitrophenyl-d-glucopyranoside were purchased from Shanghai Yuanye Biotechnology Co., Ltd. (Shanghai, China). Soluble starch was purchased from Damao Chemical Reagent Factory (Tianjin, China), 3,5-Dinitrosalicylic acid was provided by Solabao Biotechnology Co., Ltd. (Shanghai, China), and acarbose was obtained from Macklin Biochemical Technology Co., Ltd. (Shanghai, China). Ethanol, petroleum ether, dichloromethane, ethyl acetate, and n-butanol were purchased from Zhiyuan Chemical Reagent Co., Ltd. (Tianjin, China). Ultrapure water was prepared using a Milli-Q water purification system (Millipore, Bedford, MA, USA).

### 3.2. Sample and Standard Solution Preparation

The dry leaves of *A. clypearia* were extracted using 70% aqueous ethanol at a 1:10 solid:solvent ratio via ultrasound-assisted extraction for 40 min followed by drying at 60 °C for 12 h to obtain initial extract S1. A portion of the extract was diluted ten-fold with water and extracted with an equal volume of petroleum ether, dichloromethane, ethyl acetate, or n-butanol three times sequentially to obtain extracts S2, S3, S4, and S5, respectively. The solvent was removed from the remaining extract using a rotary evaporator, and the product was dried to obtain extract S6. Another portion of the extract was separated on a polyamide column (200–300 mesh) with a gradient of solvents with increasing polarities consisting of EtOH and H_2_O (20:80, 40:60, 60:40, and 80:20, *v*/*v*) to yield fractions S7, S8, S9, and S10, respectively. Next, all extracts (S1–S10) were diluted in 70% aqueous EtOH and filtered through a 0.22 μm microporous membrane to achieve the final extract solutions.

Control samples were prepared using standard references and the compounds were isolated in our laboratory. Appropriate amounts of gallic acid, ethyl gallate, myricitrin, quercitrin, gallocatechin-7-gallate, and 7-O-galloyltricetiflavan were weighed and prepared to provide control solutions at 125 μg/mL for all solutions, except 7-O-galloyltricetiflavan at 375 μg/mL. The standard solutions were then filtered through a 0.22 μm microporous membrane.

### 3.3. UPLC Method

#### 3.3.1. UPLC-ESI-Q-TOF MS Conditions

LC-MS was performed using an AB SCIEX X500R Q-TOF mass spectrometer with the AB SCIEX X500R SCIEX OS software (AB SCIEX, USA). Mass spectrometry (MS) was conducted using an electrospray ionization (ESI) ion source in the negative ion mode. The scanning range was set to 100–1200 Da, the capillary voltage was −4500 V, and the ion source temperature was 600 °C. The other optimized conditions of the ESI source were as follows: cone hole gas flow rate, 50 L/h; nebulizing gas, 500 L/h; drying gas (Gas1), 55 psi; gas curtain gas (Gas2), 55 psi; collision gas, 7 psi; declustering voltage, 80 V; scan time, 0.52 s; cumulative sampling time, 0.1 s.

Chromatography was performed using an ACQUITY UPLC (Waters, USA) system with an ACQUITY UPLC T3 C18 column (100 × 2.1 mm, 1.8 μm) at 30 °C. The mobile phase consisted of 0.1% formic acid water solution (A) and methanol (B) in the gradient elution mode as follows: 6–17% B, 0–2 min; 17–26% B, 2–15 min; 26–32% B, 15–25 min; 32–38% B, 25–40 min; 38–48% B, 40–50 min; 48–6% B, 50–55 min. The flow rate was 0.3 mL/min, the sample injection volume was 1 μL, and the detection wavelength was set to 270 nm.

#### 3.3.2. UPLC Conditions and Method Validation

UPLC was carried out as described in Section 3.3.1. The method was evaluated for precision, repeatability, and stability. Precision was analyzed using six successive injections of one sample solution (S1), repeatability was estimated using six replicates of a sample, and stability was determined at the intervals of 0, 2, 4, 6, 8, 12, and 24 h (Table S2).

### 3.4. Antibacterial Assay

Six common pathogenic bacteria, *Salmonella*, *Bacillus subtilis*, *Klebsiella pneumoniae*, *Staphylococcus aureus*, *Escherichia coli*, and *Pseudomonas aeruginosa,* were selected for the Kirby Bauer test and minimal inhibitory concentration (MIC) experiments. Prior to the experiment, the frozen bacteria were activated, purified, and diluted with the sterilized saline solution at a bacterial concentration of approximately 10^8^ CFU/mL bacterial suspension.

For the Kirby Bauer test [18], briefly, a bacterial suspension (200 μL) was placed and spread evenly on the agar medium; autoclaved filter paper disks were immersed into the extract (50 mg/mL) in advance (approximately 15 min), which were removed and dried (for approximately 30 min) after complete absorption of the drug solution. The drug-containing disks and the blank control disks were attached to the agar medium coated with a bacterial solution and incubated in a constant-temperature incubator at 37 °C for 24 h. After the end of the culture period, the diameter of the antibacterial zone was measured by the crossover method. Gentamicin (120 μg/disk) was used as a positive control. Each test was repeated in triplicate.

For the MIC test [9], sterilized Luria-Bertani (LB) broth and extracts (100 μL) were added to wells 1–9 of a 96-well plate and diluted to the concentrations of 3.12, 1.56, 0.78, 0.39, 0.20, 0.10, 0.05, 0.025, and 0.0125 mg/mL, respectively. The bacterial suspension was added to wells 1–9 sequentially; well 10 did not have any reagent and was used as the blank control. Furthermore, the extract (100 μL) was added to well 11 to serve as the negative control; the broth (100 μL) and the corresponding bacterial suspension (20 µL) were added to well 12, which was the positive control. The 96-well plate was placed in a constant-temperature incubator at 37 °C for 24 h, and 1% 2,3,5-triphenyltetrazolium chloride (TTC) solution (20 μL) was added to each well to determine the MIC value. Similarly, gentamicin was used for further antibacterial activity comparison as a standard antibiotic.

### 3.5. α-Glucosidase and α-Amylase Inhibition Assay

The α-glucosidase inhibition assay was performed as described by Franco et al. [19]. Phosphate buffer (50 μL, 100 mmol/L, pH 6.8, PBS), α-glucosidase solution (0.5 U/mL, 20 μL), and sample solution (10 μL) were added to a 96-well plate sequentially. After the reaction at 37 °C for 5 min, p-nitrophenyl-α-d-glucopyranoside (pNPG) (5 mmol/L, 20 μL) of the substrate was added, and the mixture was incubated in a 37 °C water bath for 15 min. The reaction was terminated by adding a 0.2 mol/L sodium carbonate solution (50 μL) for 15 min, and the absorbance was measured at 405 nm.

The α-amylase inhibition assay was carried out according to the procedure reported by Podsedek et al. [20] with several modifications. In a centrifugal tube, the sample (50 μL), α-amylase solution (50 μL), and phosphate-buffered solution (100 μL, pH 6.8) were mixed, to which 1% soluble starch (100 μL) was added, followed by reaction at 37 °C for 10 min. Then, 3,5-dinitrosalicylic acid (200 μL) was used to color the solution for 5 min in a 100 °C water bath, and the mixture was finally diluted to 4 mL with distilled water to detect the absorbance at 405 nm.

The inhibition rate was measured by the following equation, and the IC_50_ was calculated using GraphPad Prism 8.0 (GraphPad Software, Inc., La Jolla, CA, USA). Each test was repeated in triplicate.

$$\mathrm{inhibition}\;\mathrm{rate}\left(\%\right)=[1-\frac{\left(\mathrm{Asa}-\mathrm{Asc}\right)}{\left(\mathrm{Aea}-\mathrm{Aec}\right)}]\times 100$$

where Asa, Asc, Aea, and Aec are the absorbances of the sample group (enzyme + sample + substrate), sample control group (sample + PBS), enzyme activity group (PBS + enzyme + substrate), and enzyme blank group (substrate + PBS), respectively.

### 3.6. Spectrum–Effect Relationship Analysis

#### 3.6.1. Gray Relational Analysis (GRA)

The gray correlation analysis of six bacteria and two enzymes was performed using Deng’s correlation degree method in the gray evaluation system [21]. The antibacterial zone diameter values, the reciprocal of the IC_50_ of the α-glucosidase and α-amylase inhibition rates were used as the systematic behavioral characteristic sequences Y01, Y02, and Y03, and the quantified peak areas of the characteristic peaks of the 10 different polarity extracts were set as subsequences Xi, Xi = (xi(1), xi(2), …, xi(n)); the correlation between Y01, Y02, Y03, and Xi(k) was calculated and ranked to determine the influence of each peak area on the bio-efficacy [22].

#### 3.6.2. Partial Least Squares Regression Analysis (PLSR)

The chromatographic peak areas of 10 different polarity extracts were normalized and set as the independent variables (X), and the antibacterial zone diameter values of six bacteria and the IC_50_ values of two enzyme inhibitory activities were set as the dependent variables (Y). PLS models were established sequentially using the SIMCA 14.1 software (MKS Umetrics, Umea, Sweden). The main active compounds were screened and expressed as predicted values for the importance of each variable (VIP).

### 3.7. Verification Experiment of the Isolated Compounds

Six compounds were isolated to verify the antibacterial and antidiabetic activity of the screened components, with the separation method described in detail in the Supplementary Materials. The MIC values of the purified compounds for six bacteria were determined, and the IC_50_ values of the inhibition rate of α-glucosidase and α-amylase were tested, as described in Section 3.4 and Section 3.5.

### 3.8. Antibacterial Mechanism Prediction

The substances with the highest potential antibacterial activity, as selected on the basis of the spectrum–effect relationship analysis, were further analyzed to predict antibacterial targets and pathways using the network pharmacology method. Structure files of 7-O-galloyltricetiflavan were obtained by searching the PubChem database (http://pubchem.ncbi.nlm.nih.gov/ (accessed on 7 October 2022)), and prediction drug targets were uploaded through the PharmMapper database (http://lilab-ecust.cn/pharmmapper/ (accessed on 7 October 2022)), where the species was set to human, and default values of the other parameters were used. Six bacterial names were used as search terms in the GeneCards (https://www.genecards.org/ (accessed on 9 October 2022)) and Online Mendelian Inheritance in Man (OMIM, http://omim.org/ (accessed on 9 October 2022)) databases to retrieve antimicrobial gene targets. The intersection of the prediction drug targets and antimicrobial targets was imported into the STRING platform (https://string-db.org/ (accessed on 10 October 2022)) to construct protein–protein interaction (PPI) networks with the species set to human and the interaction score to ≥0.4.

The gene ontology (GO) function and the Kyoto encyclopedia of genes and genomes (KEGG) pathway enrichment analyses were performed. The Bioconductor module package “org.Hs.eg.db” in the R software was used to run the complete entrez ID conversion. The “colorspace,” “stringi,” “ggplot2,” “DOSE” and “clusterProfiler” modules in the R software were used to perform the GO function and KEGG pathway enrichment analysis under a filter condition of *p* > 0.05 [23]. The top 20 pathways in the KEGG and targets with a node degree number above the average value were selected to construct a “component–target–pathway–disease” network using cytoscape 3.9.1.

### 3.9. Molecular Docking of α-Glucosidase and α-Amylase

The 3D structures of the different components of *A. clypearia* were obtained from the PubChem database and were converted to the PDB format using the Open Babel software. The 3D structures of α-glucosidase (ID:3A4A) and α-amylase (ID:1OSE) were obtained from the PDB database (https://www.rcsb.org/ (accessed on 25 October 2022)) in the PDB format [15,24]. The PyMOL 2.5.1 molecular graphics system was used to remove water molecules and small molecule ligands of the proteins. The target proteins and compounds were converted to the PDBQT format using AutoDockTools 1.5.6. The docking method was performed using the Lamarckian Genetic Algorithm, and the number of docking times was set to 200.

## 4. Conclusions

In this study, 26 compounds of *A. clypearia* were identified by LC-MS, and 20 peaks with high stability and good separation were selected as characteristic peaks. The spectrum–effect relationship between the biological activity and the quantitative peak areas of different polarity extracts of *A. clypearia* were evaluated by PLSR and GRA. P15, P16, and P20 were considered the main antibacterial and antidiabetic components. Further antibacterial and antidiabetic assays of six isolated compounds of *A. clypearia* showed that the bioactivity is ranked in the order of organic acids (P4, P8) < flavonoids (P14, P17) < flavanones (P9, P15), which is consistent with the spectrum–effect relationship analysis results. In addition, network pharmacology was implemented to predict the potential targets of the best antibacterial component, and a “component–target–pathway–disease” network was constructed. The network diagram demonstrated that 7-O-galloyltricetiflavan (P15) inhibits the six bacteria by affecting the SRC, MAPK1, AKT1, HSP90AA1, HRAS, PTPN11, EGFR, LCK, RHOA, and MAPK8 targets as well as the lipid and atherosclerosis, prostate cancer and adherens junction pathways. Finally, the binding energies of the seven main components of *A. clypearia* to α-amylase and α-glucosidase were calculated through molecular docking. The binding energy (<−6 kcal/mol) of flavanones further verified the accuracy of the identification of the antidiabetic components. Moreover, the binding sites of 7-O-galloyltricetiflavan and 7,4′-di-O-galloyltricetiflavan (P16/P20) to α-amylase and α-glucosidase were analyzed. The molecule 7-galloyltricetiflavan binds to the HIS-305, TYR-151, HIS-201, HIS-299, HIS-305, and GLU-233 amino acid residues on α-amylase and to the ASP-307, LYS-156, LEU-313, PHE-314, ASN-415, ARG-315, HIS-280, and SER-304 amino acid residues on α-glucosidase; meanwhile, 7,4′-di-O-galloyltricetiflavan binds to the HIS-101, ILE-148, GLN-161, VAL-163, GLU-233, HIS-299, ASP-300, and HIS-305 amino acid residues on α-amylase and to the TYR-158, SER-241, ASP-242, GLY-309, THR-310, and PRO-320 amino acid residues on α-glucosidase. The network pharmacology and molecular docking results will contribute to the mechanism study of the active substance 7-O-galloyltricetiflavan in the future.

In summary, the relevant antidiabetic and antibacterial active substances were successfully detected using a combination of spectrum–effect relationship analysis, network pharmacology, and molecular docking, providing guidance for the subsequent development of related products as well as new analytical approaches for the evaluation of the pharmacological activity of other traditional medicine ingredients.

## Figures and Tables

**Table 1:** Identification of *A. clypearia* components.

No.	RT(min)	Formula	[M − H]^−^ (*m*/*z*)	Error (ppm)	MS^2^ Ions	Identification
1 (P1)	0.65	-	-	-	-	Unknow
2 (P2)	0.93	-	-	-	-	Unknow
3 (P3)	2.38	C_13_H_16_O_10_	331.0639	−6.3	271.0449, 211.0245, 169.0132, 151.0031, 125.0237, 89.0248, 71.0137, 59.0133	6-O-Galloylglucose
4 (P4)	2.94	C_7_H_6_O_5_	169.0133	0.8	125.0227, 79.0184	Gallic acid
5	5.04	C_13_H_16_O_9_	315.0713	1.2	169.0140, 151.0031, 123.008, 59.0133	Luteolin-5,3′-dimethylether
6 (P5)	6.78	C_19_H_20_O_12_	439.0859	−2.7	313.0564, 169.0137, 125.0238	3,5-Dihydroxyphenyl 1-O-(6-O-Galloyl-Beta-d-Glucopyranoside)
7	7.25	C_15_H_14_O_7_	305.0651	−1.5	169.0136, 125.0242	Epigallocatechin
8 (P6)	8.97	C_20_H_20_O_14_	483.0767	−0.5	271.0456, 169.0134	1,6-bis-O-galloyl-beta-d-glucose
9 (P7)	12.47	C_15_H_14_O_6_	289.0703	−1.4	151.0389, 137.0236, 109.0290, 83.0130	(−)−5,7,3′,4′,5′-pentahydroxyflavan
10 (P8)	13.58	C_9_H_10_O_5_	197.0447	1.2	169.0131, 124.0149, 78.0102	Ethyl gallate
11 (P9)	15.35	C_22_H_18_O_11_	457.0743	−4.8	305.0634, 261.0749, 219.0651, 179.0337, 137.0236, 125.0233	Gallocatechin-7-gallate
12	18.61	C_20_H_36_O_10_	435.2224	−0.1	389.2185, 227.1624, 161.0747, 113.0252, 85.0274, 71.0152	Phlorizin
13 (P10)	19.44	C_22_H_18_O_10_	441.0798	−4.2	289.0709, 151.0386, 137.0227, 125.0237	Epicatechin gallate
14 (P11)	21.34	C_22_H_18_O_11_	457.0754	−2.6	305.0633, 261.0761, 219.0646, 179.0335, 165.0185, 137.0233, 125.0232	Epigallocatechin-7-gallate
15 (P12)	23.40	C_29_H_22_O_15_	609.0868	−1.1	457.0763, 305.0659, 219.0657, 179.0353, 125.0235	Gallocatechin 7,4′-di-O-gallate
16	23.88	C_21_H_20_O_13_	479.0826	1.1	316.0214, 271.0240	Myricetin 3-galactoside
17	25.72	C_22_H_18_O_10_	441.0816	−0.1	289.0713, 151.0394, 137.0238, 125.0238	(−)-Epicatechin gallate
18 (P13)	28.33	C_21_H_20_O_12_	463.0855	−3.4	316.0200, 271.0237	Myricitrin
19 (P14)	30.81	C_21_H_20_O_12_	463.0853	−3.9	300.0259, 271.0236, 255.0291	Isoquercitrin
20 (P15)	31.74	C_22_H_18_O_10_	441.0806	−2.3	289.0709, 151.0386, 137.0227, 125.0237	7-O-galloyltricetiflavan
21 (P16)	39.30	C_29_H_22_O_14_	593.0907	−3.1	441.0780, 289.0695, 151.0389, 137.0225, 125.0236	7,4′-di-O-galloyltricetiflavan
22 (P17)	40.33	C_21_H_20_O_11_	447.0910	−2.6	300.0244, 271.0231, 255.0286, 178.9983, 151.0032	Quercitrin
23 (P18)	44.51	C_29_H_22_O_14_	593.0912	−2.3	441.0820, 289.0712, 151.0396, 137.0239, 125.0239	Catechin 7,3′-Di-O-Gallate
24 (P19)	46.05	C_29_H_22_O_14_	593.0910	−2.6	441.0806, 289.0708, 151.0395, 137.0234, 125.0240	Catechin 7,4′-di-O-gallate
25 (P20)	48.33	C_29_H_22_O_14_	593.0917	−1.5	441.0783, 289.0690, 151.0387, 137.0223, 125.0234	7,4′-di-O-galloyltricetiflavan
26	51.09	C_36_H_26_O_18_	745.1021	5.9	593.0926, 441.0817, 289.0702, 137.0243	Trifucodiphlorethol A

**Table 2:** Antibacterial zone diameter results.

Batch	Antibacterial Zone Diameter (mm)					
	*Salmonella*	*Bacillus subtilis*	*Klebsiella pneumoniae*	*Escherichia coli*	*Staphylococcus aureus*	*Pseudomonas aeruginosa*
S1	9.78 ± 0.72	12.06 ± 0.44	10.33 ± 0.56	10.96 ± 0.06	10.56 ± 0.09	10.15 ± 0.25
S2	9.41 ± 0.68	9.29 ± 0.78	12.21 ± 0.95	10.94 ± 0.72	11.51 ± 0.66	10.96 ± 0.06
S3	8.00 ± 0.40	10.51 ± 0.42	9.42 ± 0.54	8.47 ± 0.21	8.75 ± 0.56	8.92 ± 0.71
S4	10.97 ± 0.45	13.14 ± 0.58	13.29 ± 1.10	14.74 ± 0.49	10.66 ± 0.24	13.01 ± 0.38
S5	8.12 ± 0.15	7.14 ± 0.56	8.22 ± 0.27	8.87 ± 0.51	8.58 ± 0.10	7.97 ± 0.09
S6	7.28 ± 0.16	7.28 ± 0.26	7.05 ± 0.10	6.80 ± 0.29	7.11 ± 0.08	6.35 ± 0.39
S7	7.71 ± 0.04	6.96 ± 0.04	6.59 ± 0.04	7.15 ± 0.13	8.60 ± 0.29	6.94 ± 0.06
S8	8.17 ± 0.25	12.74 ± 0.80	9.86 ± 0.26	11.50 ± 0.60	10.64 ± 0.27	9.98 ± 0.09
S9	8.37 ± 0.29	13.03 ± 0.26	10.76 ± 0.66	11.29 ± 0.36	9.87 ± 0.48	10.66 ± 0.25
S10	9.55 ± 0.10	13.14 ± 0.47	10.96 ± 0.19	11.30 ± 0.26	10.32 ± 0.22	10.90 ± 0.36
gentamicin	25.36 ± 0.48	26.05 ± 0.33	22.97 ± 0.78	22.21 ± 0.75	22.02 ± 0.45	23.17 ± 0.48

**Table 3:** Inhibition of α-amylase and α-glucosidase.

Batch	IC_50_ (μg/mL)	
	α-Amylase	α-Glucosidase
S1	258.93 ± 23.60	2.41 ± 0.23
S2	160.67 ± 12.00	2.01 ± 0.16
S3	394.17 ± 38.34	3.84 ± 0.05
S4	180.73 ± 18.09	2.88 ± 0.34
S5	596.93 ± 23.61	3.60 ± 0.21
S6	-	99.21 ± 7.07
S7	-	122.57 ± 0.72
S8	1193.00 ± 113.32	10.24 ± 0.62
S9	250.10 ± 17.53	2.36 ± 0.09
S10	261.23 ± 36.10	1.65 ± 0.05
acarbose	65.59 ± 6.59	0.69 ± 0.14

**Table 4:** GRA results.

Peak	*Salmonella*		*Bacillus subtilis*		*Klebsiella pneumoniae*		*Escherichia coli*		*Staphylococcus aureus*		*Pseudomonas aeruginosa*		α-Amylase		α-Glucosidase	
	cor.	rank	cor.	rank	cor.	rank	cor.	rank	cor.	rank	cor.	rank	cor.	rank	cor.	rank
P1	0.771	14	0.755	16	0.760	16	0.762	16	0.764	16	0.759	16	0.821	5	0.811	4
P2	0.766	17	0.753	18	0.757	18	0.757	18	0.759	19	0.755	18	0.806	10	0.791	9
P3	0.764	18	0.754	17	0.757	17	0.758	17	0.762	17	0.757	17	0.823	3	0.804	6
P4	0.787	12	0.768	15	0.778	13	0.779	14	0.786	11	0.779	13	0.788	13	0.789	11
P5	0.763	19	0.743	20	0.754	19	0.753	19	0.759	18	0.754	19	0.752	19	0.753	19
P6	0.805	9	0.792	10	0.794	10	0.799	10	0.806	6	0.797	10	0.763	18	0.764	17
P7	0.768	16	0.771	14	0.774	15	0.777	15	0.764	15	0.774	15	0.785	14	0.778	12
P8	0.756	20	0.749	19	0.751	20	0.746	20	0.755	20	0.750	20	0.752	20	0.763	18
P9	0.775	13	0.778	12	0.779	12	0.785	12	0.772	13	0.780	12	0.800	11	0.772	14
P10	0.817	2	0.828	3	0.827	2	0.835	2	0.820	2	0.829	2	0.815	8	0.789	10
P11	0.771	15	0.774	13	0.776	14	0.781	13	0.768	14	0.776	14	0.800	12	0.771	15
P12	0.812	4	0.835	1	0.817	4	0.820	4	0.815	3	0.820	4	0.784	15	0.778	13
P13	0.807	8	0.803	9	0.805	9	0.813	9	0.804	7	0.807	9	0.784	16	0.765	16
P14	0.810	5	0.804	8	0.808	8	0.817	5	0.808	4	0.811	7	0.823	4	0.794	8
P15	0.820	1	0.830	2	0.827	1	0.836	1	0.823	1	0.831	1	0.826	2	0.806	5
P16	0.810	6	0.823	4	0.815	5	0.815	6	0.803	8	0.816	5	0.821	6	0.835	1
P17	0.788	11	0.784	11	0.785	11	0.793	11	0.785	12	0.788	11	0.774	17	0.751	20
P18	0.815	3	0.819	6	0.821	3	0.821	3	0.807	5	0.821	3	0.812	9	0.801	7
P19	0.802	10	0.813	7	0.809	7	0.815	7	0.795	10	0.811	8	0.830	1	0.835	3
P20	0.809	7	0.822	5	0.814	6	0.814	8	0.802	9	0.815	6	0.820	7	0.835	2

**Table 5:** MIC values of isolated compounds for six bacteria.

Compound	MIC (mg/mL)					
	*Salmonella*	*Bacillus subtilis*	*Klebsiella pneumoniae*	*Escherichia coli*	*Staphylococcus aureus*	*Pseudomonas aeruginosa*
Gallic acid (P4)	1.56	0.39	1.56	1.56	1.56	3.12
Ethyl gallate (P8)	0.39	0.39	0.20	0.78	0.78	0.78
gallocatechin-7-gallate (P9)	0.39	0.10	0.10	0.10	0.20	0.78
7-O-galloyltricetifavan (P15)	0.20	0.10	0.10	0.20	0.20	0.39
Myricitrin (P13)	1.56	0.78	1.56	0.78	1.56	1.56
Quercitrin (P17)	3.12	1.56	1.56	0.78	1.56	1.56
gentamicin	0.006	<0.006	0.012	0.012	0.012	0.012

**Table 6:** Binding energy calculation results.

	Binding Energy(kcal/mol)	
	α-Amylase	α-Glucosidase
Gallic acid (P4)	−4.16	−4.70
Ethyl gallate (P8)	−4.48	−4.87
Gallocatechin-7-gallate (P9)	−6.27	−7.21
7-O-galloyltricetifavan (P15)	−6.69	−7.45
7,4′-di-O-galloyltricetiflavan (P16/P20)	−6.32	−7.89
Myricitrin (P13)	−5.52	−7.82
Quercitrin (P17)	−5.87	−7.44
crystallized ligand	−5.07	−5.20

## Data Availability

Data is contained within the article and supplementary material.

## Supplementary Materials

The following supporting information can be downloaded at: https://www.mdpi.com/article/10.3390/molecules28031329/s1, Table S1. Quantitative peak areas of the different polarity extracts of *A. clypearia*; Table S2. Method validation results of UPLC; Table S3. MIC values of the *A. clypearia* extracts; Table S4. IC_50_ values of the six isolated compounds for α-glucosidase and α-amylase; Figure S1. Total ion current of *A. clypearia*; Figure S2. Antibacterial zones of the different polarity extracts; Isolation method and spectroscopic data of the isolated compounds. References [25,26] are cited in Supplementary Materials.
