# Supplementary material for: Discovery, Validation, and Target Prediction of Antibacterial and Antidiabetic Components of Archidendron clypearia Based on a Combination of Multiple Analytical Methods
Source: Molecules. 2023 Jan 30;28(3):1329. doi: 10.3390/molecules28031329 (PMC9919075; doi:10.3390/molecules28031329)
Supplement: Supplementary file 1 [file molecules-28-01329-s001.zip › molecules-2126539-supplementary.pdf]

Figure S1. Total ion current of *A. clypearia*.

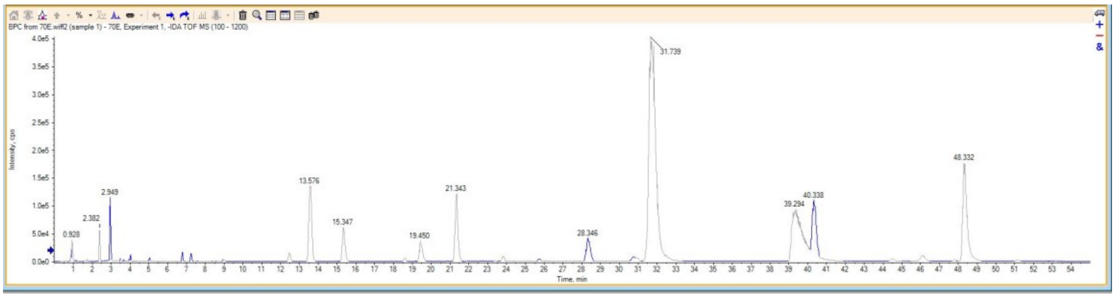

Figure S2. Antibacterial zone of different polarity extracts.

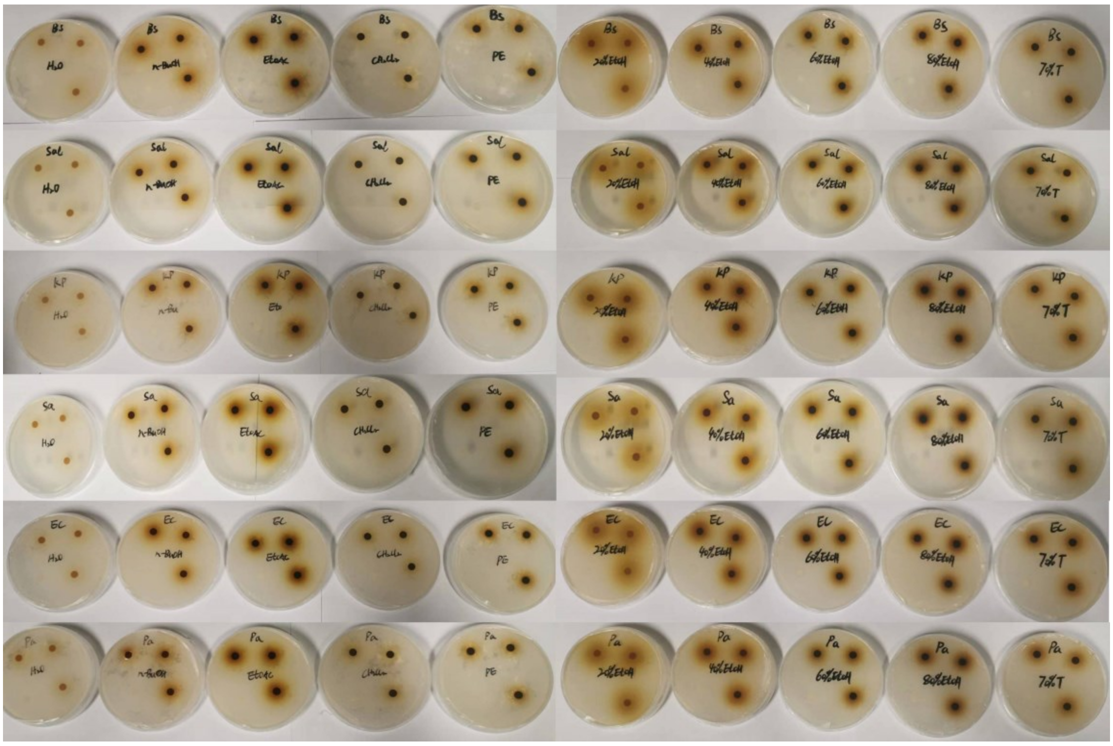

Table S1. Quantitative peak areas of different polarity extracts of *A. clypearia*.

Peak	Retention time (min)	S1	S2	S3	S4	S5	S6	S7	S8	S9	S10
P1	0.82	82.86	24.75	15.49	0.01	71.72	377.44	0.01	0.01	0.01	0.01
P2	1.19	116.00	22.72	22.80	0.01	287.59	476.74	40.61	19.29	0.01	0.01
P3	2.40	90.85	0.01	0.01	0.01	367.51	154.04	0.01	71.51	0.01	0.01
P4	2.93	1086.01	66.44	203.41	1751.52	2430.00	1108.64	13748.24	106.53	129.33	35.46
P5	6.74	52.65	0.01	0.01	32.35	306.63	16.41	378.59	79.47	0.01	0.01
P6	8.73	88.20	0.01	0.01	138.54	257.20	67.57	163.96	446.59	15.01	0.01
P7	12.04	55.67	10.10	0.01	142.39	31.02	0.01	0.01	780.41	0.01	0.01

P8	13.15	648.17	92.23	2872.28	972.67	52.53	0.01	6586.68	46.31	113.05	74.30
P9	14.63	331.38	106.40	54.00	698.76	39.91	0.01	0.01	1887.82	20.96	0.01
P10	18.34	144.69	68.05	19.95	342.71	52.23	0.01	0.01	180.53	731.56	118.82
P11	20.16	579.69	167.32	90.10	1262.40	86.37	0.01	0.01	3549.26	0.01	0.01
P12	21.85	83.48	46.17	0.01	171.31	87.55	0.01	0.01	505.54	379.46	201.38
P13	26.00	434.33	153.28	67.05	824.10	223.21	0.01	1154.72	1859.36	193.74	180.88
P14	27.95	175.63	106.56	37.61	359.39	173.15	0.01	416.05	993.21	53.92	0.01
P15	28.61	5039.51	3798.64	1395.18	9929.17	605.20	0.01	0.01	6544.58	6220.48	598.54
P16	35.65	3496.74	3897.21	760.47	5784.94	1138.35	0.01	0.01	0.01	10347.93	19192.71
P17	37.05	1020.22	439.11	246.98	1920.70	171.38	0.01	2064.04	5108.76	0.01	0.01
P18	42.10	49.31	84.12	16.42	139.57	15.97	0.01	0.01	0.01	619.14	113.83
P19	43.75	123.02	149.05	37.98	295.91	57.40	0.01	0.01	29.63	515.16	692.83
P20	46.45	2192.89	2482.20	512.48	3691.65	758.01	0.01	0.01	0.01	6729.72	12345.21

Table S2. Method validation results of UPLC.

Peaks	RSD (%)		
	precision	repitability	stability
P1	0.26	3.68	1.11
P2	0.66	1.93	1.92
P3	2.12	3.20	2.82
P4	0.35	2.27	1.28
P5	0.54	1.72	1.37
P6	0.38	2.87	0.79
P7	1.59	1.89	1.95
P8	0.80	2.30	2.13
P9	1.02	2.15	1.27
P10	3.11	3.77	1.03
P11	0.29	0.80	0.29

P12	2.24	3.22	3.60
P13	0.80	3.05	0.75
P14	1.66	3.17	2.15
P15	0.31	0.98	0.37
P16	0.32	1.78	0.59
P17	0.95	1.49	1.73
P18	1.52	1.25	2.74
P19	2.04	2.65	3.76
P20	0.25	2.68	0.58

Table S3. MIC values of the *A. clypearia* extracts.

Batch	MIC (mg/mL)					
	Salmonella	*Bacillus subtilis*	*Klebsiella pneumoniae*	*Escherichia coli*	*Staphylococcus aureus*	*Pseudomonas aeruginosa*
S1	1.56	0.78	0.78	0.20	0.78	0.78
S2	1.56	1.56	1.56	1.56	1.56	0.78
S3	1.56	0.78	0.78	0.39	3.12	>3.12
S4	0.78	0.78	0.39	0.20	1.56	0.39
S5	>3.12	>3.12	1.56	>3.12	>3.12	>3.12
S6	>3.12	>3.12	>3.12	>3.12	>3.12	>3.12
S7	>3.12	3.12	>3.12	>3.12	>3.12	>3.12
S8	1.56	0.78	0.39	0.20	1.56	0.39
S9	1.56	0.39	0.39	0.20	1.56	0.39
S10	0.78	0.78	0.39	0.20	0.78	0.39

Table S4. IC_{50} values of the six isolated compounds for α-glucosidase and α-amylase

Compound	IC50（μg/ml）	
	α-Amylase	α-glucosidase
Gallic acid (P4)	>800	>800
Ethyl gallate (P8)	>800	97.99±8.85
gallocatechin-7-gallate (P9)	383.93±24.38	13.56±1.07
7-O-galloyltricetifavan (P15)	177.50±17.72	2.87±0.13
Myricitrin (P13)	>800	50.81±1.67
Quercitrin (P17)	>800	48.87±3.15

Extraction and isolation method of six compounds:

The dried leaves and twigs of *A. clypearia* (50 g) were extracted using 2000 mL of 70% EtOH for 40 min by ultrasonication to obtain 20 g of crude extract. The sample was chromatographed on a polyamide column (200–300 mesh) with a gradient of increasing polarity of a solvent system comprising EtOH and H2O (20:80, 40:60, 60:40, and 80:20, v/v) to yield four fractions (A–D). Fraction A was separated using ODS gel column chromatography (CC) and eluted with MeOH–H2O (20:80, v/v) to give compounds 1 (18mg, P4) and 2 (15mg, P8). Fraction B was further fractionated by polyamide gel CC (200–300 mesh) with a step gradient of EtOH-H2O (from 20:80 to 50:50, v/v) to yield fractions B1 and B2. Fraction B1 was then passed over ODS gel CC and eluted with mixtures of MeOH–H2O (30:70, v/v) to yield compounds 3 (8mg, P13) and 4 (10mg, P17); fraction B2 was separated on ODS gel CC with MeOH–H2O (20: 80, v/v) as an eluent and then purified by reversed-phase (RP) preparative high-performance liquid chromatography (HPLC) (1 mL/min) to obtain compound 5 (12mg, P9) and compound 6 (24mg, P15).

Spectroscopic data of the isolated compounds are below:

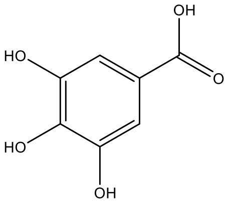

Compound 1: white amorphous solid; ESI-MS: m/z 169 [M − H]−. According to the data and compared with the standard sample, compound 1 was determined to be gallic acid.

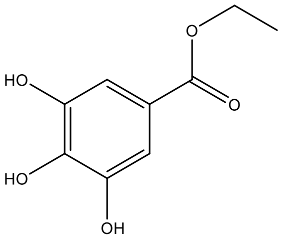

Compound 2: white amorphous solid; ESI-MS: m/z 197 [M − H]−. According to the data and compared with the standard sample, compound 2 was determined to be ethyl gallate.

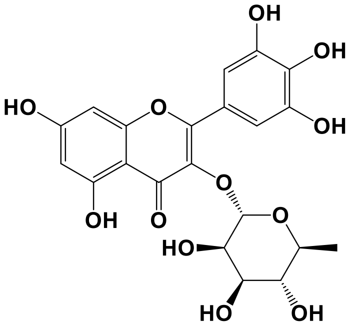

Compound 3: yellow amorphous solid; ESI-MS: m/z 463 [M − H]−. According to the data and compared with the standard sample, compound 4 was determined to be myricitrin.

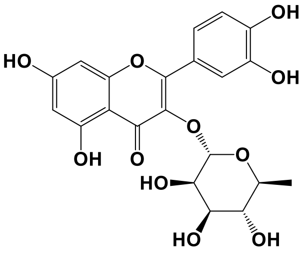

Compound 4: yellow amorphous solid; ESI-MS: m/z 447 [M − H]−. According to the data and compared with the standard sample, compound 6 was determined to be quercitrin.

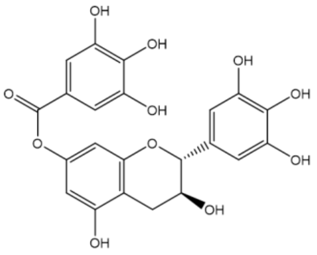

Compound 5: white amorphous solid; ESI-MS: m/z 457 [M − H]−, $[\alpha]^{20}_{D}$ +18.87 (c 0.5, MeOH), **^{1}H NMR (500 MHz, DMSO)** δ 7.05 (s, 2H), 6.26 (d, J = 0.6 Hz, 2H), 6.19 (d, J = 2.3 Hz, 1H), 6.12 (d, J = 2.3 Hz, 1H), 4.58 (d, J = 6.6 Hz, 1H), 3.89 (q, J = 6.4 Hz, 1H), 2.68 (dd, J = 16.4, 5.1 Hz, 1H), 2.47 (d, J = 7.2 Hz, 1H). **^{13}C NMR (126 MHz, DMSO)** δ 166.47, 158.14, 157.10, 151.79, 147.80, 147.76, 141.41, 134.63, 131.52, 111.05, 107.83, 107.62, 102.64, 102.34, 83.20, 67.81, 29.09. According to the data and reported literature values [1, 2], compound 3 was determined to be gallocatechin-7-gallate.

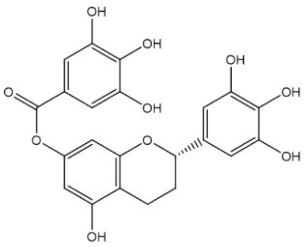

Compound 6: white amorphous solid; ESI-MS: m/z 441 [M − H]−, $[\alpha]^{20}_{D}$ −4.22 (c 0.5, MeOH), **^{1}H NMR (500 MHz, DMSO)** δ 7.05 (s, 2H), 6.32 (s, 2H), 6.18 (d, J = 2.3 Hz, 1H), 6.10 (d, J = 2.3 Hz, 1H), 4.79 (dd, J = 9.9, 2.2 Hz, 1H), 2.65 – 2.53 (m, 2H), 2.11 – 2.01 (m, 1H), 1.90 – 1.76 (m, 1H). **^{13}C NMR (126 MHz, DMSO)** δ 166.47, 158.09, 157.99, 151.64, 147.92, 147.76, 141.16, 134.56, 133.60, 120.49, 111.07, 109.00, 107.10, 102.87, 102.47, 79.02, 30.65, 21.14. According to the data and reported literature values [1], compound 5 was determined to be 7-O-galloyltricetifavan.

References

1. Kang, J.; Liu, C.; Wang, H.; Li, B.; Li, C.; Chen, R.; Liu, A. Studies on the bioactive flavonoids isolated from Pithecellobium clypearia Benth. *Molecules* **2014**, *19*, 4479-4490, doi:10.3390/molecules19044479.
2. Maldini, M.; Montoro, P.; Hamed, A.I.; Mahalel, U.A.; Oleszek, W.; Stochmal, A.; Piacente, S. Strong antioxidant phenolics from Acacia nilotica: Profiling by ESI-MS and qualitative-quantitative determination by LC-ESI-MS. *Journal of Pharmaceutical and Biomedical Analysis* **2011**, *56*, 228-239, doi:10.1016/j.jpba.2011.05.019.

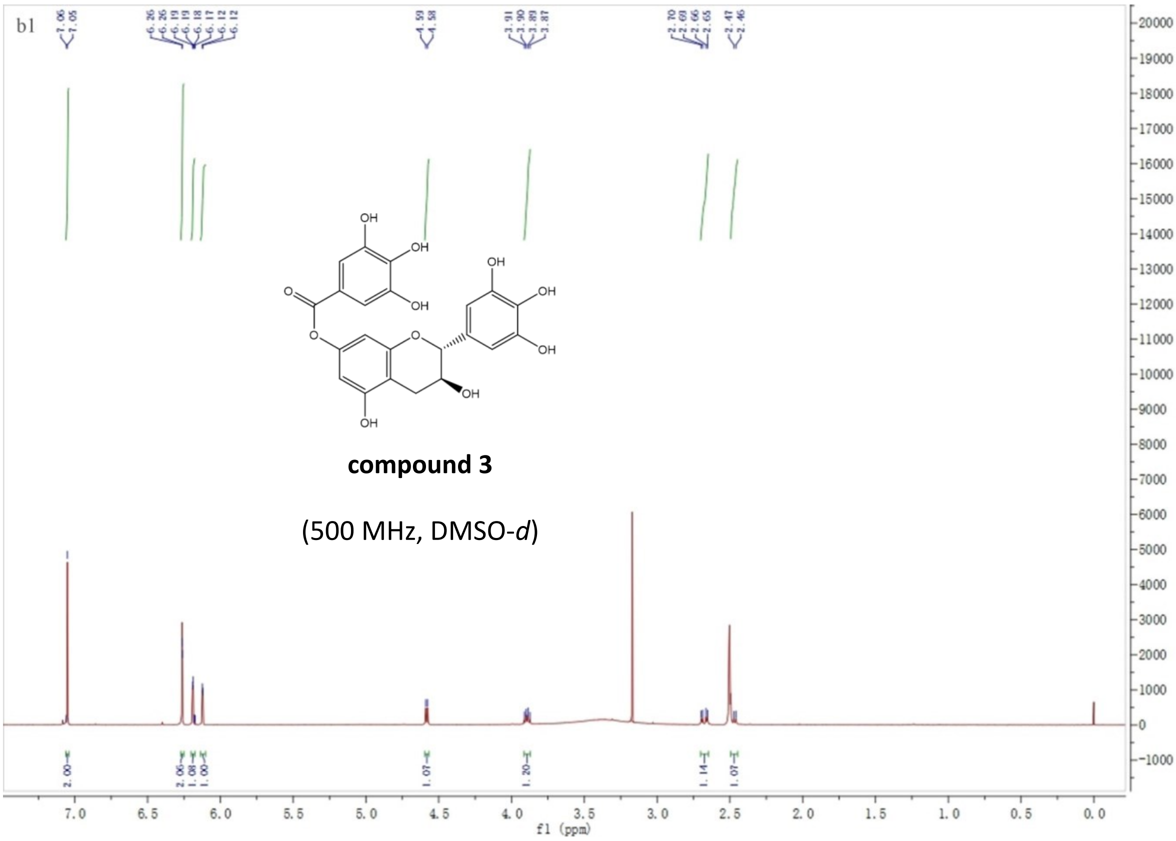
b1
7.06
7.05
6.26
6.26
6.19
6.19
6.18
6.17
6.12
6.12
4.59
4.58
3.91
3.90
3.89
3.87
2.70
2.69
2.66
2.65
2.47
2.46
compound 3
(500 MHz, DMSO-d)
2.00
2.06
1.08
1.00
1.07
1.20
1.14
1.07
f1 (ppm)

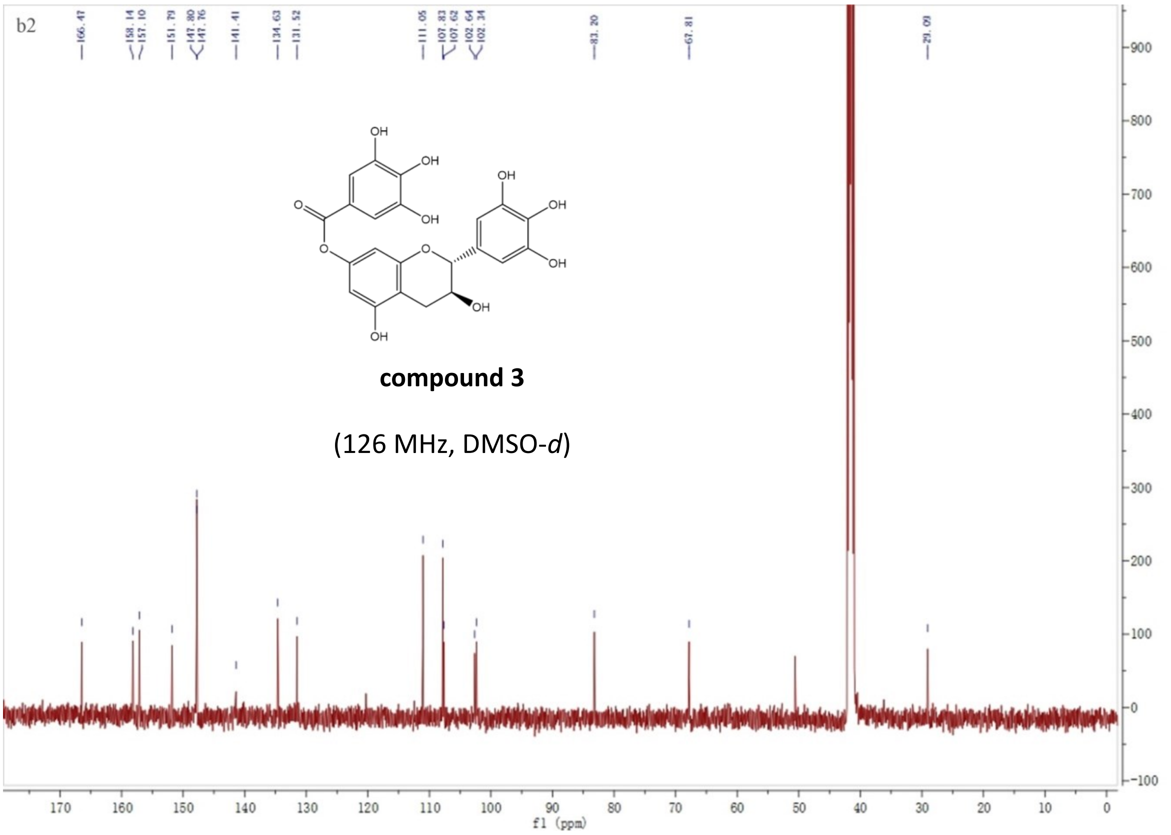
b2
166.47
158.14
157.10
151.79
147.80
147.76
141.41
134.63
131.52
111.05
107.83
107.62
102.64
102.34
83.20
67.81
29.09
compound 3
(126 MHz, DMSO-d)
f1 (ppm)

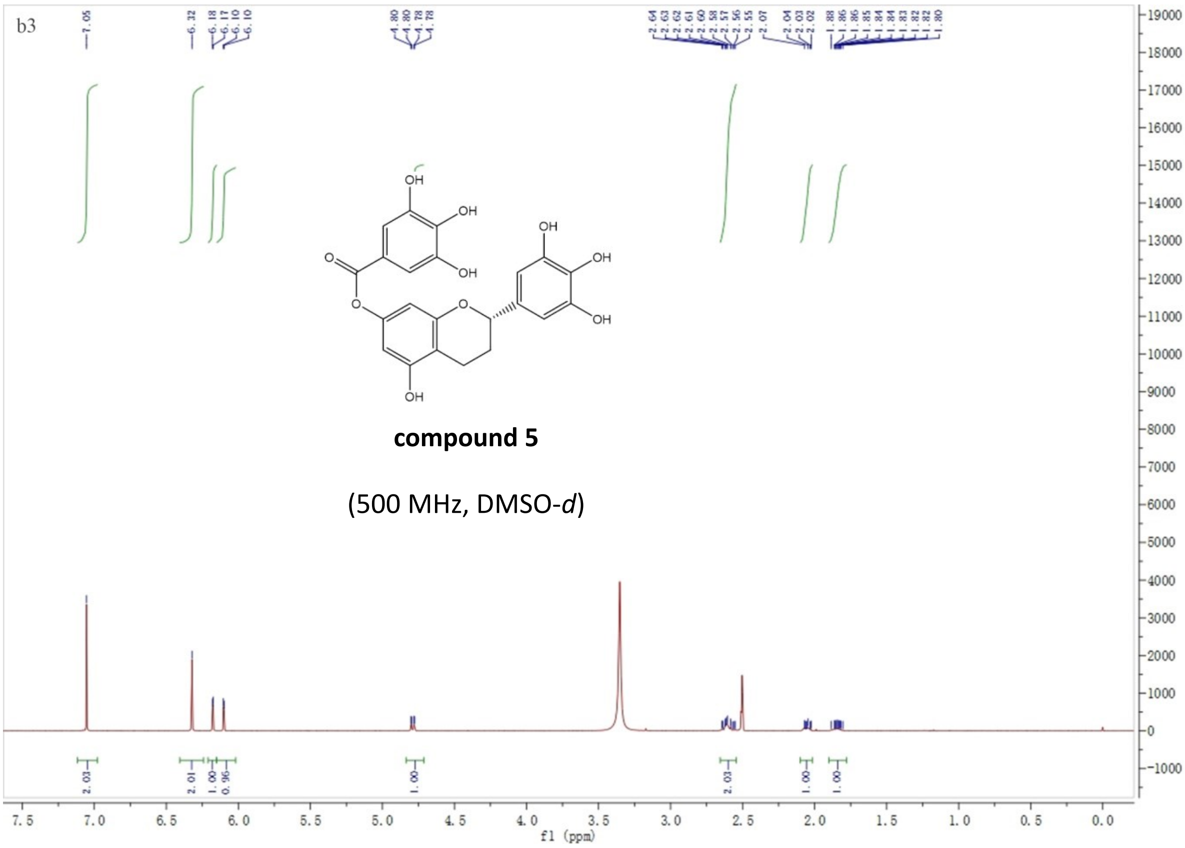
b3
7.05
6.32
6.18
6.17
6.10
6.10
4.80
4.80
4.78
4.78
2.64
2.63
2.62
2.61
2.60
2.58
2.57
2.56
2.56
2.55
2.07
2.04
2.03
2.02
1.88
1.86
1.86
1.85
1.85
1.84
1.84
1.83
1.83
1.82
1.82
1.80
OH
OH
OH
O
O
O
OH
OH
OH
OH
compound 5
(500 MHz, DMSO-d)
2.03
2.01
1.00
0.96
1.00
2.03
1.00
1.00
f1 (ppm)

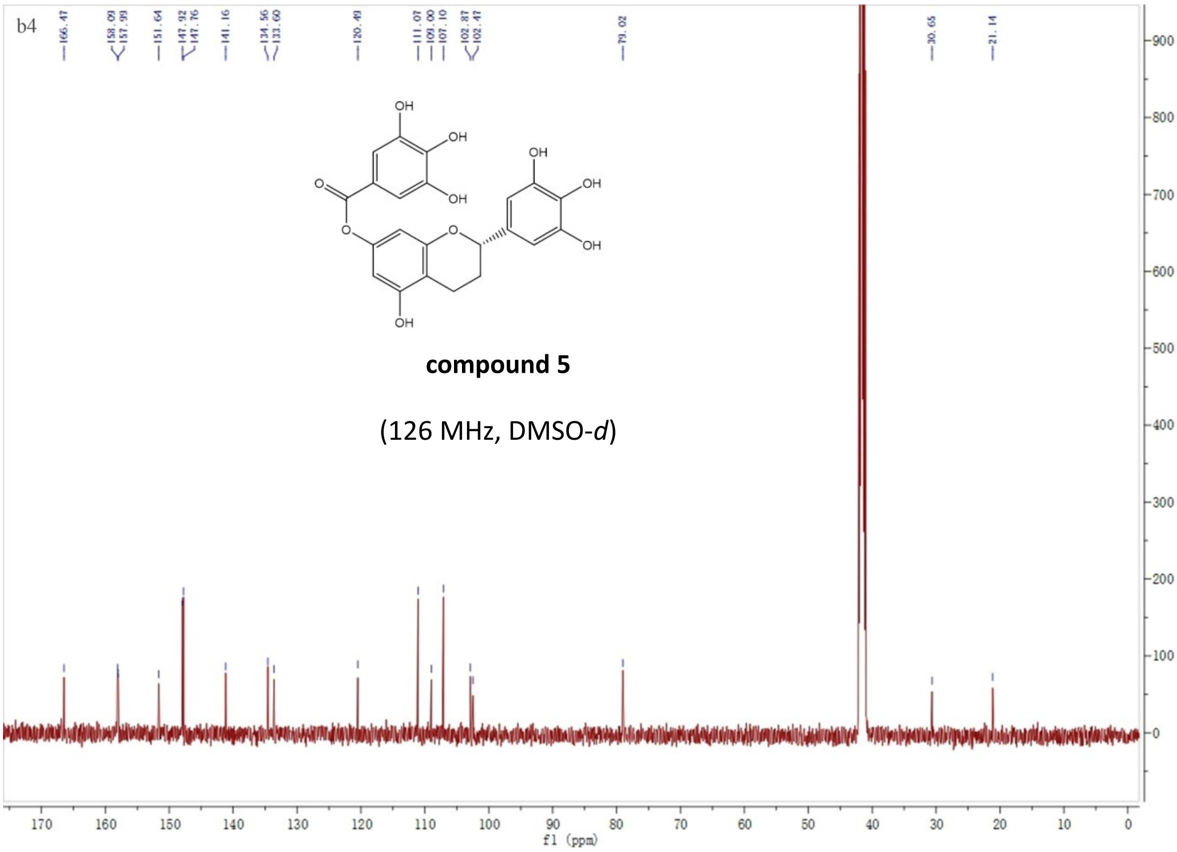
b4
166.47
158.09
157.99
151.64
147.92
147.76
141.16
134.56
133.60
120.49
111.07
109.00
107.10
102.87
102.47
79.02
30.65
21.14
OH
OH
OH
O
O
O
OH
OH
OH
OH
compound 5
(126 MHz, DMSO-d)
f1 (ppm)
